# Impacts of Road Traffic Network and Socioeconomic Factors on the Diffusion of 2009 Pandemic Influenza A (H1N1) in Mainland China

**DOI:** 10.3390/ijerph16071223

**Published:** 2019-04-05

**Authors:** Bo Xu, Huaiyu Tian, Clive Eric Sabel, Bing Xu

**Affiliations:** 1 Ministry of Education Key Laboratory for Earth System Modeling, Department of Earth System Science, Tsinghua University, Beijing 100084, China; xu-b15@mails.tsinghua.edu.cn; 2 Joint Center for Global Change Studies, Beijing 100875, China; 3 State Key Laboratory of Remote Sensing Science, College of Global Change and Earth System Science, Beijing Normal University, Beijing 100875, China; tianhuaiyu@gmail.com; 4 Department of Environmental Science, Aarhus University, 4000 Roskilde, Denmark; cs@envs.au.dk

**Keywords:** 2009 H1N1 pandemic, highway network, socioeconomic factors, spatiotemporal transmission, gravity model, network node centrality, spatial autoregressive model, mainland China

## Abstract

The 2009 pandemic influenza virus caused the majority of the influenza A virus infections in China in 2009. It arrived in several Chinese cities from imported cases and then spread as people travelled domestically by all means of transportation, among which road traffic was the most commonly used for daily commuting. Spatial variation in socioeconomic status not only accelerates migration across regions but also partly induces the differences in epidemic processes and in responses to epidemics across regions. However, the roles of both road travel and socioeconomic factors have not received the attention they deserve. Here, we constructed a national highway network for and between 333 cities in mainland China and extracted epidemiological variables and socioeconomic factors for each city. We calculated classic centrality measures for each city in the network and proposed two new measures (*SumRatio* and *Multicenter Distance*). We evaluated the correlation between the centrality measures and epidemiological features and conducted a spatial autoregression to quantify the impacts of road network and socioeconomic factors during the outbreak. The results showed that epidemics had more significant relationships with both our new measures than the classic ones. Higher population density, higher per person income, larger *SumRatio* and *Multicenter Distance*, more hospitals and college students, and lower per person GDP were associated with higher cumulative incidence. Higher population density and number of slaughtered pigs were found to advance epidemic arrival time. Higher population density, more colleges and slaughtered pigs, and lower *Multicenter Distance* were associated with longer epidemic duration. In conclusion, road transport and socioeconomic status had significant impacts and should be considered for the prevention and control of future pandemics.

## 1. Introduction

The 2009 Influenza A (H1N1) pandemic posed one of the most serious global public health challenges in recent years [1]. With the ease and speed of global travel in the 21st century, the world is now a global village in terms of epidemic transmission [2]. The Influenza A (H1N1) virus can transmit from humans to humans by direct body contact or respiratory droplets. The infectious disease can spread widely as people carrying pathogens travel and commute between cities by multiple means of transport. Thus, intercity travel is important for the diffusion of viruses [3,4,5].

Among transport modes, road traffic should not be neglected. In the United States, the amount of people who travel daily along the interstate highways during the influenza season is larger than 3.8 million, which is over twice those who travel by air [6]. In mainland China, road traffic conveyed around 28 billion passengers in 2009, accounting for 93% of total passengers, much more than trains and airlines [7]. The mode choice was determined not only by Chinese people’s choice preference for travel but also by the features of various travel modes. Airlines are the fastest mode, but they were relatively expensive in China in 2009, so they mainly served domestic business and government officials, although they have become much cheaper nowadays. Railways were mainly used for occasional long-distance travelling [8] (e.g., the Spring Festival travel rush in China [9]), whereas highways and freeways were more often used for daily and weekly short-distance commuting [10]. Though more and more people today choose the high-speed railway for their daily trips in China, this was unavailable in 2009. National highways in China are funded and constructed by the central government and are vital connections between provinces and cities, without the collection of tolls, whereas tolls on expressways are quite high, meaning car and buses tend to avoid them [11]. Thus, passenger transportation on national highways well reflected the nationwide general highway transportation system in China in 2009.

Road traffic is considered to play an important role in the spread of infectious diseases. City traffic can be modelled as a road network to help simulate the transmission of airborne diseases in a city [12]. National highways and interprovincial freeways were the foremost path for Severe Acute Respiratory Syndrome (SARS) being carried by people from Beijing to neighboring areas in 2003 [13]. The transmission pattern of dengue in Cambodia has been found to be related to the network of national roads, where traffic allowed human carriers to move [14]. The high-density road network is an important explanatory factor to the dengue epidemic in 2014 in Guangzhou city and Foshan city in China [5]. For the highly pathogenic avian influenza, national highways were one of the most important predictive environmental variables for the infection risk in 2004 in China [11,15], and the number of poultry outbreaks had a positive association with the road density in Indonesia during 2003–2008 [16]. In the United States, the genetic distances between seasonal influenza A (H1N1) viruses were found to correlate with the distances along those parts of the interstate highways most used by commuters, along which only 1% of commutes occurred over distances of more than 242 kilometers, and the role of this road-based travel in facilitating influenza transmission should not be neglected [6]. Research on the spread of the 2009 influenza A (H1N1) virus in the Republic of Korea showed that the cumulative number of new cases increased in the direction along a highway line [17]. Highways, expressways, and county roads showed their importance to the transmission of the same virus between city and town [1]. It has also been shown that counties in China intersected by national highways and freeways, but not railways, were significantly associated with the spread of this virus [10]. Moreover, the confined space in buses and cars are more limited than that in trains, and the vehicles are usually more crowded and filled with stagnant air, which may facilitate infection to fellow travelers [13] in spite of air filters as an effort to reduce potential virus aerosol concentrations.

In addition to ongoing urbanization which draws people from rural to urban areas, spatial heterogeneity [17] and social disparities in a basic infrastructure and economy are also stimuli to human migration across regions, which facilitates the spatial diffusion of infectious diseases [18] and may lead to regional inequalities of disease progression and burdens [19]. A previous study found that income, railway capacity, per person gross domestic product, and urban rate played roles in the interregional transmission of SARS at the province level in China [20]. Urbanization has been reported to partially affect the transmission of seasonal influenza in U.S. cities [21]. Other socioeconomic factors like population density, illiteracy, and unemployment were significantly associated with the mortality from the 1918 influenza pandemic in Chicago [19]. As for the 2009 influenza pandemic in China, it has been suggested that the spatial patterns of public places impacted the mixing and commuting of people, which then influenced virus transmission within Changsha city [22]; another study suggested that, apart from domestic travel, population density was related to the spread of viruses at the county level [10].

In this study, we constructed a national highway network including 333 prefecture-level cities in mainland China and extracted three epidemiological variables (cumulative incidence, onset week, and duration of epidemics) and 14 socioeconomic factors (related to population, income level, medical condition, livestock breeding, and school education) in each city. We calculated four classic centrality measures (degree, betweenness, closeness, and eigenvector centrality) for each city in the network and proposed two new measures (*SumRatio* and *Multicenter Distance*). We first evaluated the correlation between the centrality measures and epidemiological features, and then used spatial autoregressive models to investigate the impacts of road network and socioeconomic factors on the diffusion of the 2009 influenza pandemic.

## 2. Materials and Methods

### 2.1. Data

The suspected and laboratory-confirmed cases of influenza A (H1N1) used in this study were obtained from the surveillance system of the Chinese Center for Disease Control and Prevention, with dates ranging from week 19 (May) in 2009 to week 53 (December) in 2010. Every data entry represents a case with attributes, such as the date of onset, date of birth, and address together with a corresponding administrative area number. MATLAB [23] and ArcGIS Desktop [24] were used to extract information concerning the cumulative incidence which is the cumulative number of daily reported cases during the above time period divided by the population at risk, the onset week which is defined here as the week when the first case in a city was reported, and the duration which is the time from onset to the first epidemic peak, all in a certain prefecture-level city.

Statistical data for prefecture-level cities in 2009 were collected from the China Economic and Social Development Statistical Database (http://tongji.cnki.net), including highway passenger capacity, urbanization ratio (denoted as Urban ratio), population density (PopDensity), per person gross domestic product (PGDP), average wage of employees (Income), number of hospitals per 10,000 population (Hospital), number of hospital beds per 1000 population (Hos-bed), number of doctors per 10,000 population (Doctor), number of colleges (College), number of middle schools (MidSchool), number of primary schools (PriSchool), number of college students per 100 population (CollegeStu), number of middle school students per 100 population (MidSchoolStu), number of primary school students per 100 population (PriSchoolStu), and number of slaughtered pigs (Pig).

### 2.2. National Highway Network

The network of national highways in China consists of nodes (representing prefecture-level cities) and links (representing national highway lines). Every link is connected to two nodes, to which the two corresponding prefecture-level cities are connected by a section of national highways.

There are seventy national highway lines in China. We calculated the actual distance between any pair of adjacent prefecture-level cities along the national highways and assigned a unique number to each city. Two symmetric matrices (matrix **A** and **D1**) were constructed. Matrix **A** is an adjacency matrix. If city i and j are adjacent along a national highway, then the value of element **A** (i, j) is one, else it is zero. Matrix **D1** is an adjacent distance matrix and element **D1** (i, j) is the actual distance between city i and j if they are adjacent to each other along national highways (if not, **D1** (i, j) equals zero). An unsymmetrical matrix **D2** was generated from matrix **D1** using the Floyd algorithm [25] to store the shortest distances between cities along the national highways.

### 2.3. Network Node Centrality

#### 2.3.1. Classic Centrality Measures

We calculated four common network node centrality measures for each city node, including the degree, which is the number of nodes directly adjacent to it; the betweenness, which is the number of shortest paths that pass through the node; the closeness, which is the sum of the shortest distances (geodesic paths) between that node and all other nodes in the network; and the eigenvector centrality, which is a measure of the influence of a node in a network [26]. These node centralities are calculated with the igraph package [27] in the R program [28].

#### 2.3.2. Newly Proposed Centrality Measures

Inspired by the method of Dominant Flow Analysis [29], we propose a new node centrality measure called *SumRatio*, which is a synthetic indicator combining the number of passengers and the spatial distance, measuring the global importance of a node to all others in a network. Higher values of *SumRatio* relate to more frequent exchanges of people and economic trade, which makes it easier for viruses to spread among individuals. *SumRatio* can be calculated as follows.

(1) $$G\left(i,j\right)=m\frac{N_{i}^{\beta }N_{j}^{\delta }}{d_{ij}^{\gamma }},$$

(2) $$Gp\left(i,j\right)=\frac{G\left(i,j\right)}{\sum_{j=1\sim n,\;j\neq i}G\left(i,j\right)},$$

(3) $$SumRatio\left(j\right)=\underset{i=1\sim n,\;i\neq j}{\sum }Gp\left(i,j\right),$$

where G(i,j) represents the gravity coefficient between cities i and j; *N* is the outflow highway passenger capacity; *d* is the shortest distance between two cities along national highways; and m, β, δ, and γ are constants. Gp(i,j) represents the ratio of gravity coefficient between cities i and j to the sum of gravity coefficients between city i and any other cities in the network and measures the relative importance of city j to city i. SumRatio(j) is the sum of the gravity coefficient ratio, which measures the integrated importance of city j to all other cities in the network. The two matrices (matrix G and Gp) are generated using a gravity model [30,31] based on the demographic data. Matrix G is a symmetric matrix while Gp is unsymmetrical [32]. n is the total number of cities in the national highway network (n=333). m, β, and δ are set to be 1, 1, and 1, respectively, as estimated by Reference [32]. γ is set to be 1 according to the result of Reference [31]. *N* can be estimated as follows: Because the “highway passenger capacity” figures found in the statistical yearbook consisted of three parts, including the highway passenger capacity within a city, the inflow passenger capacity, and outflow passenger capacity of a city, we assumed that the inflow and outflow passenger capacities of a city are equal to each other and that they both could compose “the passenger capacity between cities”. Among the 104,096 nonzero values of the distance stored in matrix **D2**, more than 98% were larger than 200 km. Hence, we assumed that the highway passenger capacity within a city can be represented by the number of passengers transported within the range of a circle whose center is a certain city with a radius of 100 km. It has also been reported that the highway passenger volume occurred within a distance shorter than 100 km accounted for more than 90% of the total highway passenger volume. Therefore, we assumed that the highway passenger capacity within a city accounted for 90% of the total highway passenger volume and that the outflow highway passenger capacity accounted for 5%.

**Figure 1:**
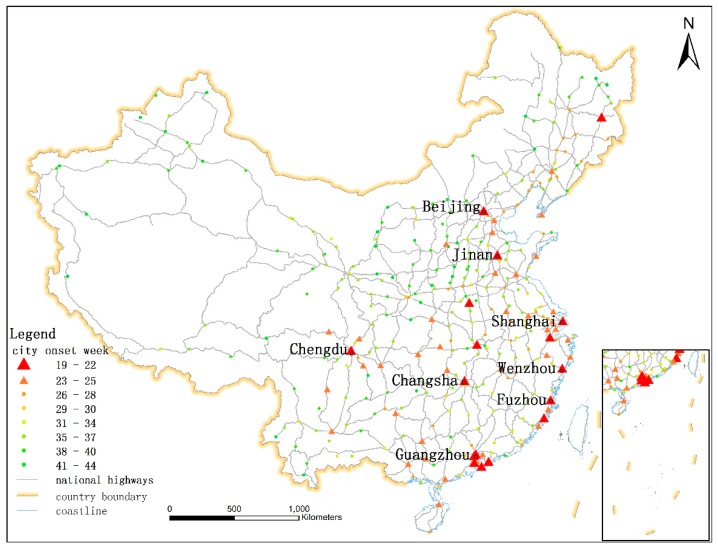
The spatial distribution of the epidemic onset week in each city: The points and triangles represent cities. Different colors correspond to different onset weeks. The gray lines represent national highways. The eight center cities are labeled with their names.

We propose another new node centrality measure called *Multicenter Distance*, which refers to the shortest distance along national highways from a certain city to one of the eight center cities (Chengdu, Jinan, Beijing, Guangzhou, Shanghai, Fuzhou, Wenzhou, and Changsha city) where the epidemics arrived the earliest (before May 22, 2009). All the first cases in these eight cities were imported into China by planes from other countries. The spatial locations of the eight cities are shown in Figure 1. The *Multicenter Distance* of city i (SD_i_) is calculated as follows.

SD_i_ = min {D_ij_, i = all cities, j = center cities}, (4)

where i = 1, 2, 3, … , *n*; *n* is the total number of cities; j = 1, 2, 3, … , *m*; *m* indicates the number of central cities; and D_ij_ is the distance along the national highways between a certain city i and a center city j.

### 2.4. Statistical Analysis

A correlation analysis was performed to assess the associations between disease variables (cumulative incidence, onset week, and duration from onset to the first epidemic peak) and highway network node centralities (adjacent degree, betweenness, closeness, eigenvector centrality, *SumRatio*, and *Multicenter Distance*) using the Pearson correlation coefficient and to figure out the significant explanatory variables for the following spatial regression analysis. To investigate the quantitative relationships between epidemic characteristics (e.g., cumulative incidence, onset week, and duration) and socioeconomic factors, including Urban ratio, PopDensity, PGDP, Income, Hospital, Hos-bed, Doctor, College, MidSchool, PriSchool, CollegeStu, MidSchoolStu, PriSchoolStu, and Pig, as well as the network structure parameters of the city vertices calculated above and to quantify the contribution of road transportation and the spatial distance to the spread of the influenza virus, we used spatial autoregressive models [33] performed in MATLAB. In the models, epidemic characteristics were set to be response variables, while the selected network node centrality measures and socioeconomic factors were included as explanatory variables. We were able to collect the records of all these variables in 273 cities where Influenza A (H1N1) cases had been reported.

Before the regression analysis, we calculated the variance inflation factor (VIF) of each explanatory variables to detect collinearity.

A general version of the spatial model including both the spatial lag term and a spatially correlated error structure is shown as

*y* = *ρW*_1_*y* + *Xβ* + *μ* *μ* = *λW*_2_*μ* + *ε* *ε* ~ *N* (0, *σ*^2^*I_n_*), (5)

where *y* denotes an *n* × 1 vector of response variable observations collected at *n* cities; *X* is an *n* × *k* matrix representing explanatory variables; *ε* is an *n* × 1 vector of normally distributed (with constant variance *σ^2^*) stochastic disturbances; *n* is the total number of cities; *k* is the number of explanatory variables incorporated into the regression model; *W_1_* and *W_2_* are *n* × *n* spatial weight matrices, usually containing contiguity relations; the parameter *ρ* is a coefficient on the spatially lagged response variable ($W_{1}y$); *β* represents the regression coefficients to be estimated to reflect the influence of the explanatory variables *X* on the variation of the response variable *y*; the parameter *λ* is a coefficient on the spatially correlated error *μ*; and *I_n_* is an *n × n* unit matrix.

## 3. Results

### 3.1. Development of the Influenza A (H1N1) Pandemic in Mainland China in 2009

**Figure 2:**
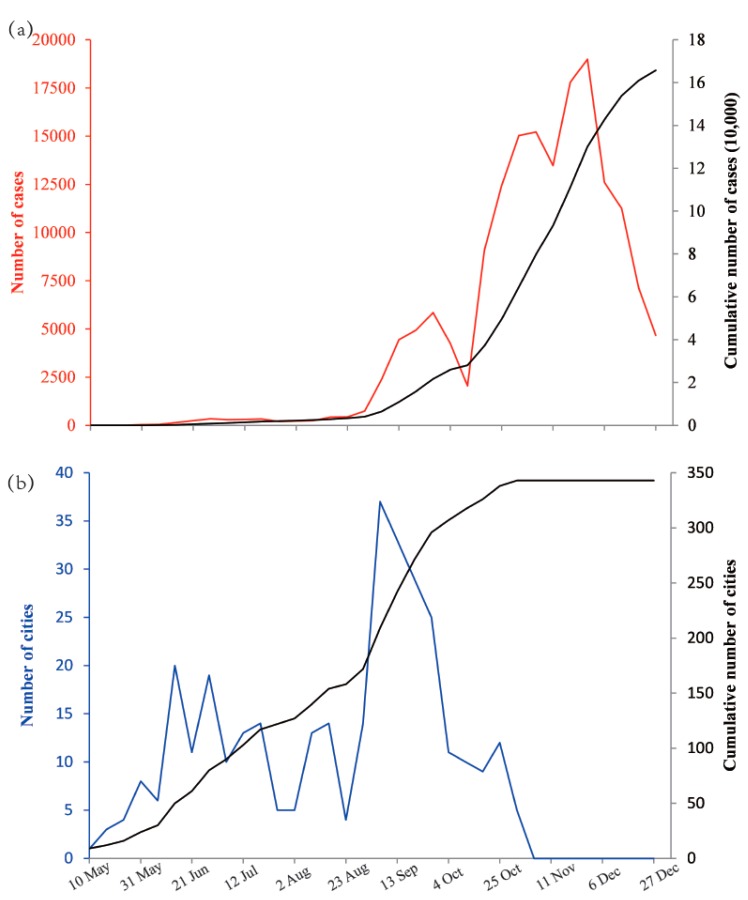
The development of the influenza pandemic in mainland China in 2009: (**a**) The weekly incidence (red) and cumulative incidence (black) from May 10 to December 27 and (**b**) the weekly number (blue) and cumulative number (black) of cities where cases were reported from May 10 to December 27.

The earliest onset was week 19 and the latest was week 44 in 2009. The earliest epidemic peak in all cities arrived in week 35, and the last one arrived in week 52. As shown in Figure 2a, the weekly incidence curve rose slowly from May 10 to August 30. Then, it ascended quickly and arrived in the first peak (5853 cases) on September 27. During the period from October 18 to December 20, the weekly incidence was larger than 7000 cases and reached the second peak (18,989 cases) on November 29. As shown in Figure 2b, the cumulative number of cities where cases had ever been reported increased moderately before August 23. Thereafter, it climbed up quickly and decelerated after September 27. It reached a plateau in December 1, when nearly all Chinese cities had been exposed to the emerging influenza virus.

### 3.2. Characteristics of the National Highway Network

**Figure 3:**
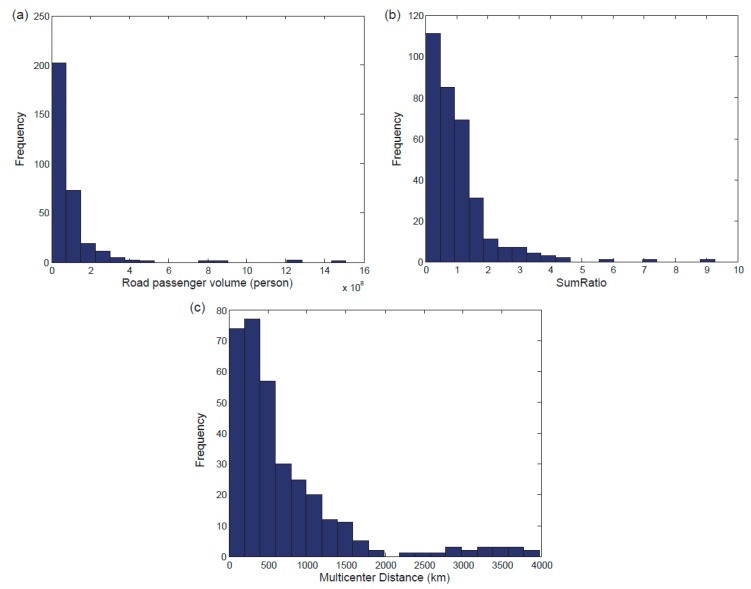
The characteristics of the national highway network: Histograms of the road passenger volumes (**a**), *SumRatio* (**b**), and *Multicenter Distance* (**c**) of all the cities in the national highway network.

The national highway network had a mean degree of 3.06, an average clustering coefficient of 0.102, a diameter of 26, and an average path length of 10.645. In Figure 3a–c, histograms of the road passenger volumes, *SumRatio*, and *Multicenter Distance* of all the cities in the national highway network are displayed, respectively.

### 3.3. Correlation Analysis

As shown in Table 1, each of the two newly proposed centrality measures (*SumRatio* and *Multicenter Distance*) was significantly correlated with all three epidemiological features (cumulative incidence, onset week, and duration). While the correlations between classic centrality measures (degree, betweenness, and eigenvector centrality, except for closeness centrality) and any of the three epidemiological features were not statistically significant. In addition, *SumRatio* and *Multicenter Distance* performed even better than closeness centrality. *Multicenter Distance* was positively correlated with the cumulative incidence (*r* = 0.258, *p* < 0.001) and onset week (*r* = 0.320, *p* < 0.001) but negatively correlated with the duration from onset to the first epidemic peak (*r* = −0.369, *p* < 0.001). A positive correlation between closeness centrality and onset week (*r* = 0.208, *p* < 0.001) was identified, as well as a negative association between closeness and duration (*r* = −0.126, *p* < 0.05). *SumRatio* was positively associated with the cumulative incidence (*r* = 0.354, *p* < 0.001) and duration (*r* = 0.265, *p* < 0.001), but negatively associated with the onset week (*r* = −0.348, *p* < 0.001). The Pearson correlation coefficients between the number of slaughtered pigs and cumulative incidence, onset week, and duration of epidemics were −0.096 (*p* = 0.175), −0.259 (*p* < 0.001), and 0.271 (*p* < 0.001), respectively. Therefore, the number of pigs would not be taken as an explanatory variable of the regression model when the cumulative incidence was taken as the response variable.

### 3.4. Spatial Autoregressive Analysis

Based on the results of correlation analysis, we incorporated three network node centrality measures (closeness, *SumRatio*, and *Multicenter Distance*) into spatial autoregressive models as explanatory variables.

The VIF of each explanatory variable except for closeness centrality (17.128) was less than 10 (Table 2), so all the explanatory variables but closeness were able to enter the regression model.

When we took the cumulative incidence as the response variable in the spatial autoregressive model, the spatial dependence coefficient ρ of the response variable was statistically significant (*ρ* = 0.104, *p* =0.001). The cumulative incidence can increase 0.205%, −0.140%, 0.286%, 0.141%, 0.147%, 0.190%, and 0.326%, with a 1% increase in PopDensity, PGDP, Income, Hospital, CollegeStu, *SumRatio*, and *Multicenter Distance*, respectively (Table 2). When we took the onset week as the response variable in the spatial autoregressive model, the spatial dependence coefficient of the response variable was statistically significant (*ρ* = 0.216, *p* < 0.001). Onset week can be delayed by 0.413% and 0.510% when PopDensity and Pig decreased by one percent, respectively (Table 2).When we took the duration from onset to the first epidemic peak as the response variable in the spatial autoregressive model, the spatial dependence coefficient of the response variable was also statistically significant (*ρ* = 0.237, *p* < 0.001). Duration can be lengthened by 0.803%, 0.541%, 0.472%, and −0.277%, with a 1% increase in PopDensity, College, Pig, and *Multicenter Distance*, respectively (Table 2). 

## 4. Discussion

In this study, we evaluated the impacts of road traffic and other socioeconomic factors on the nationwide spread of influenza A (H1N1) across China in 2009. We constructed a national highway network and proposed two new centrality measures: *SumRatio* and *Multicenter Distance*. Both new measures were significantly correlated with all three epidemiological features in question (cumulative incidence, onset week, and epidemic duration). Based on the positive association between onset week and *Multicenter Distance*, we understand the transmission process of influenza A (H1N1) as follows: At the very beginning, internationally infected individuals quickly arrived in the eight center cities and other cities in eastern China with frequent international communications [34], most of which are spatially distant from each other, after long distance travel by plane [6]. By this means the virus was quickly introduced into new regions. This pattern was important for virus spread in the early stage of an epidemic, especially before strict control measures were taken [13]. The virus then diffused over short-range connections as infectious individuals moved to neighboring areas by ground-based transport along highways [6]. The epidemic followed the classic distance decay theory, starting earlier in the cities which are closer to their nearest center cities, and this was partly supported by the findings in Reference [22].

To verify our discoveries and explanations quantitatively and to acquire quantitative relationships between road traffic, as well as socioeconomic factors and the spread of influenza A (H1N1), we conducted spatial regression analyses. When the cumulative incidence was taken as a response variable in the spatial autoregressive model, the results could be interpreted as follows: People had more chances to contact each other and transmit infectious diseases with a higher base probability in cities of a higher population density [21,22,35], which resulted in a higher cumulative incidence at the end of epidemics. The income level in these cities was also higher [21], and more workers would be attracted to come. It has been previously reported that the density of medical facilities was not significantly associated with the arrival time of the first confirmed influenza A (H1N1) case in each Chinese county in 2009 [10]. Our findings elaborate on this, showing that the density of hospitals has a significant effect on the cumulative incidence at the city level. As we know, cities with more (high-level) hospitals would attract more patients seeking medical treatment [20]. Moreover, many symptomatic patients gathered in hospitals where the space was confined, and cross infection and nosocomial infection by means of virus droplet and aerosol could be facilitated [36], which would also result in a higher incidence. Previous research [37] reported that people between 5–24 years old were most affected by the 2009 influenza A (H1N1) in China. Therefore, our positive association between the proportion of college students and cumulative incidence is consistent with previous works. Cities with a higher *SumRatio* were more likely to be traffic hubs in the national highway network, and the cumulative incidence would be higher. The larger the *Multicenter Distance* of a city, the lower the medical treatment level would be, and the cumulative incidence could not be reduced quickly and would remain relatively high. As for cities with a high GDP, they would tend to conduct stronger intervention measures to control the infectious disease, reducing the cumulative incidence. When the onset week was taken as a response variable in the spatial regression, we found that the population density and the number of pigs had negative effects on the epidemic onset in cities. A higher population density meant that the corresponding city was more central and it might have been more likely to have a large airport or train station, and then, the imported cases which could start an epidemic in the city might arrive earlier. The “first” reported case may not be the true first person who was infected by the influenza virus in a given city. It is possible that an infected individual arrived in a city and infected others around him/her, but the public health authority did not receive an infection report, and then, the epidemic started silently and developed freely. Thus, the “first” case was not announced to the public until the underlying infected population had taken place. As the reservoir of the 2009 pandemic H1N1 influenza virus, pigs can carry this virus, can transmit it to human beings, and may be a magnifier of the population of virus and potentially infected people. Hence, the “first” case would be reported earlier in a city where more pigs were fed. When the duration from onset to the first epidemic peak was taken as the response variable in the spatial regression, the results can be interpreted as follows and be supported by the finding in Reference [21] that the periods of epidemics in smaller cities are shorter than those in larger cities. A city with a high population density would have a high proportion of susceptible people who are fuel for epidemics [38,39], which would prolong the duration of an epidemic. It was reported that school students were more susceptible to the 2009 influenza A (H1N1) than other age groups in China [37] and that schools were high-risk areas for influenza outbreaks and should be the focus for prevention [22,40]. Therefore, the result that the number of colleges was positively associated with the epidemic duration is consistent with the real situation. Moreover, cities with a larger value of *Multicenter Distance* would have a smaller population, and the number of susceptible people would also be less. As a result, the epidemic duration would be shorter. Intervention measures of public health authorities in China could shorten the epidemic duration, but these measures were taken upon human beings instead of pigs, which played a role of viral reservoir. The virus might spill over from pigs to the human population continuously, which would prolong the epidemic period. It may be an explanation for the significantly positive association between the number of slaughtered pigs and epidemic duration. In addition, it has been reported recently that the presence of airports and railway stations in prefecture-level cities did not have significant impacts on epidemic duration [41]. Our results complement previous findings and reveal that both road transport and socioeconomic factors had a significant influence on epidemic duration.

Among all means of transport, herein we emphasize the role of road transportation in the spatial diffusion of the 2009 influenza A (H1N1) virus in mainland China. It has been reported recently that both aviation and road travel have a significant association with the epidemic arrival day in each prefecture-level city during the whole viral diffusion period, but the role of rail travel was only significant after August 1st 2009 [41]. Although air travel is a significant factor in the global spread of the influenza virus, short-range daily travel on the ground is more important at the regional scale [6], which our results support. To the best of our knowledge, most Chinese people do not travel by air very often (at least in 2009, except for businessmen). Airplanes bring virus carriers to areas that are far from each other and disease-free before landing, which may lead to a spatially random dispersal of viral pathogens. In daily commuting, an infectious individual is likely to infect anyone with whom he/she is in contact, with the probability of infection associated with spatial distance and time length of contact, which may result in a spatially structured viral population [6]. In our spatial regression analyses, the spatial dependence coefficient *ρ* of three epidemiological features are all significant and positive, which means that the epidemic in a city was positively correlated with those in neighboring cities and indicates that road travel would be more important than air travel for the spatial transmission of the 2009 influenza A (H1N1) virus in China. Unlike some countries in Europe and America which experienced two infection peaks of influenza during 2009, China had only one autumn-winter wave in 2009, which could be attributed to the strict prevention and control strategies conducted by China at the early stage of the pandemic [34]. For example, fever screenings performed in airports and railway stations could be expected to detect most symptomatic passengers with a fever, but the expected efficiency of fever screenings in long-distance highway passenger stations should be much lower because a bus is allowed to pick up passengers along the way and they need not gather in a specifically designated station before boarding and because a considerable number of highway passengers would choose private vehicles. Therefore, it is much more difficult to restrict the diffusion of influenza virus by road travel. In this respect, the role of highway passenger transport in dispersing viruses spatially is more important than air and railway modes.

We also suggest that the socioeconomic conditions themselves not only have direct impacts on the development of an influenza pandemic but also indirectly exert an influence by stimulating human domestic travel and by changing the patterns of human aggregation, which is congruent with previous research [10,19,21]. It has been reported that people in a wealthy region travel farther than people in a relatively poor region in China due to the difference in basic facilities or people’s living standard, and a pandemic emerging in less developed regions might diffuse more slowly [42]. Interestingly, health disparities of people in less developed and developed areas in China might be reduced by the interaction of epidemiological and socioeconomic factors in the face of a newly emerging influenza pandemic, especially in the early stages. For instance, although medical treatment in developed areas is better than that in less developed areas, the epidemic arrival time would be earlier in developed areas with a longer epidemic duration. With the reduction of airfares, the opening of more domestic airlines, and the promotion and popularization of high-speed trains in China, the time cost of the journey is greatly decreased in recent years, and passengers tend to think more about the attractiveness of certain places which are mostly determined by the places’ socioeconomic and environmental conditions and think less about the length of trip distance when they choose destinations for travel. Therefore, the impact of spatial distance on the diffusion of infectious diseases may become smaller in the future, while that of socioeconomic factors will be more and more important.

It is interesting to note that the number of newly reported cases peaked at a time point later than the peak of the number of newly affected cities (Figure 2). Our explanations of this finding is that before week 34 (until 23 August) in 2009, the number of cases was quite small and increased very slowly (255 cases per week); the number of affected cities increased steadily (10 cities per week), which was most likely to be driven by the sporadic importation of overseas cases into China, because the majority of new cases before week 30 (until 26 July) were imported cases [34]. From week 35 to week 44 (until 1 November), the increase in the number of new cases sped up significantly (6126 cases per week) and reached its first peak on 27 September, and the large amount of existing cases greatly facilitated the invasion of the virus into unaffected cities; hence, the number of affected cities increased more quickly (18 cities per week) than in the prior period. This could be explained by two possible reasons. The first may be that almost all the new cases were local cases after week 36 (until 6 September), which meant the community transmission of the virus within a city had started, so the number of weekly newly affected cities arrived in its peak on 6 September and the rate of increase dropped afterwards. The second reason could be that the Chinese government took strict prevention and control measures before September 2009, but the measures were relaxed from September [34], which could also partly explain the sharp rise in the number of new cases and newly affected cities at the end of August and the beginning of September. The curve of new cases did not rise continuously but dropped to a local minimum in early October, which may be explained by the reduced case reporting during Chinese National Holidays [37] and could further enlarge the time delay between its peak and the peak of the number of newly affected cases. From week 45 to week 53 (until 31 December), no city was newly affected because almost all prefecture-level cities (except for Yushu City in Qinghai Province) in mainland China had already reported cases in the prior period, and the increasing velocity of the number of new cases was nearly twice of that in the earlier period.

There are some limitations in this study. Although the trend of case reporting was consistent with that of the percentage of respiratory specimens tested positive for the influenza virus, the number of reported cases was much less than the real size of infected individuals [34]. It was observed globally in many influenza outbreaksthat not all cases were reported to the medical services. Therefore, data from virological and serological surveillance should be taken into consideration for further information. Additionally, even though there were several socioeconomic factors which were not significantly associated with all three epidemiological features, there still existed other effective factors that were not included in this research, such as climate conditions. As an ecological study, the results of this study cannot provide evidence for causal relationships but merely suggest probable associations. In future research, a meta-population epidemiological model driven by road travel should be constructed to investigate the diffusion process of the 2009 influenza A (H1N1) under the effects of socioeconomic factors.

## 5. Conclusions

In summary, our study provides two major contributions to the literature. First, we propose two new node centrality measures for a national highway network: *SumRatio* and *Multicenter Distance*, and both perform better than classic node centralities for statistically significant correlations with epidemiological features. Second, we conclude that road transport network and socioeconomic factors had significant impacts on the diffusion of the 2009 influenza A (H1N1) virus at the city level in mainland China and that both should be candidates for the prevention and control of future influenza pandemics.

## Figures and Tables

**Table 1:** The statistical significance of the correlations between epidemiological features and network node centrality measures and the number of pigs slaughtered.

EF	Deg1	Deg2	Deg3	Betw	Close	Eigen	SumR	McDis	Pig
**CumInc**	NS	NS	NS	NS	NS	NS	0.354 ***	0.258 ***	NS
**Onset**	NS	NS	NS	NS	0.208 ***	NS	−0.348 ***	0.320 ***	−0.259 ***
**Duration**	NS	NS	NS	NS	−0.126 *	NS	0.265 ***	−0.369 ***	0.271 ***

Abbreviations: EF, epidemiological features; CumInc, cumulative incidence; Deg1, degree in 1 step; Deg2, degree in 2 steps; Deg3, degree in 3 steps; Betw, betweenness centrality; Close, closeness centrality; Eigen, eigenvector centrality; SumR, *SumRatio*; McDis, *Multicenter Distance*; Pig, number of pigs slaughtered; NS, not significant. *, **, and ***: The correlation coefficients are significant at the 0.05 level, at the 0.01 level, and at the 0.001 level, respectively.

**Table 2:** The variance inflation factor (VIF) of explanatory variables and the regression coefficients of spatial autoregressive models.

	VIF	Coefficient		
		CumInc ^a^	Onset week	Duration
Spatial dependence				
*ρ*		0.104 ***	0.216 ***	0.237 ***
*λ*		0.177	0.055	0.292 **
Effects				
const		−0.071 **	0.625 ***	0.300 ***
Urban ratio	2.644	−0.001	−0.036	−0.014
PopDensity	2.375	0.205 *	−0.413 *	0.803 ***
PGDP	4.047	−0.140 *	−0.152	−0.126
Income	3.246	0.286 ***	−0.112	0.042
Hospital	1.416	0.141 **	0.160	−0.136
Hos-bed	5.370	−0.047	−0.180	0.290
Doctor	3.364	−0.019	−0.034	−0.008
College	6.281	−0.001	−0.277	0.541 **
MidSchool	9.418	−0.060	0.281	−0.581
PriSchool	7.165	−0.056	−0.070	0.235
CollegeStu	3.917	0.147 **	−0.130	−0.022
MidSchoolStu	2.398	−0.084	−0.218	0.179
PriSchoolStu	3.017	0.137	0.331	−0.123
Pig	2.980		−0.510 **	0.472 **
closeness ^b^	17.128			
SumRatio	1.815	0.190 ***	−0.015	−0.131
McDistance	7.084	0.326 ***	0.158	−0.277 **
$R^{2}$		0.423	0.462	0.454
${\bar{R}}^{2}$		0.390	0.416	0.407
Log-likelihood		388.970	121.302	122.541
$W_{1},\;W_{2}$		w^c^, $w^{2}$	$w^{2},\;w$	$w,\;w^{2}$

^a^ CumInc is the same as that in Table 1. ^b^ The closeness centrality was not included in the models as an explanatory variable because its VIF was larger than 10. ^c^ w is the adjacent matrix A in the Materials and Methods Section. *, **, and *** The regression coefficients are significant at the 0.05 level, at the 0.01 level, and at the 0.001 level, respectively.

## Data Accessibility

The data and analysis code for this study have been made publicly available under the MIT license at the GitHub repository, https://github.com/BoXu123/H1N1RoadSocioeco.
